# Epidemiology of Latent Tuberculosis in Rheumatic Immune-Mediated Inflammatory Diseases—Study of 1117 Patients and Descriptive Literature Review

**DOI:** 10.3390/jcm13247546

**Published:** 2024-12-11

**Authors:** Joy Selene Osorio-Chávez, David Martínez-López, Carmen Álvarez-Reguera, Virginia Portilla, José Manuel Cifrián, Santos Castañeda, Iván Ferraz-Amaro, Ricardo Blanco

**Affiliations:** 1 Department of Pneumology, Hospital Universitario Marqués de Valdecilla, IDIVAL, Immunopathology Group, Avda. Valdecilla s/n., 39008 Santander, Spain; joyselene.osorio@scsalud.es (J.S.O.-C.); josemanuel.cifrian@scsalud.es (J.M.C.); 2 Department of Rheumatology, Hospital Universitario Marqués de Valdecilla, IDIVAL, Immunopathology Group, Avda. Valdecilla s/n., 39008 Santander, Spain; david200999@hotmail.com (D.M.-L.); alvarezreguera@gmail.com (C.Á.-R.); virgiportilla@hotmail.com (V.P.); 3 Rheumatology, Hospital Universitario La Princesa, IIS-Princesa, 28006 Madrid, Spain; scastas@gmail.com; 4 Department of Rheumatology, Hospital Universitario de Canarias, 38320 Santa Cruz de Tenerife, Spain; iferrazamaro@hotmail.com

**Keywords:** latent tuberculosis infection, rheumatic immune-mediated inflammatory diseases, tuberculin skin test, IGRA

## Abstract

**Background/Objectives:** Patients with rheumatic immune-mediated diseases (rheumatic-IMID) and latent tuberculosis (LTBI) are at an increased risk of developing active tuberculosis (TB); therefore, screening is recommended before starting biological treatment. The aims of this study were as follows: (i) to assess the prevalence of LTBI, (ii) to determine the importance of using a booster test in TST-negative patients, (iii) to compare the tuberculin skin test (TST) with the interferon-gamma release assay (IGRA), (iv) to perform a review of the prevalence of LTBI. **Methods:** A cross-sectional hospital study was performed, including patients diagnosed with rheumatic-IMID who underwent a TST and/or IGRA during the period 2016–2020. If the first TST was negative, a new TST (booster) was performed. **Results:** A total of 1117 patients were included. The overall prevalence of LTBI was estimated to be 31.7% (95% confidence interval, 29.74–33.66). The LTBI prevalence ranged from 38.5% for vasculitis to 14% for sarcoidosis. The booster test was positive in 22.9% of 817 patients with a negative or indeterminate IGRA. The IGRA was positive in 3.8% of 793 patients with a negative booster.The adjusted Cohen’s kappa coefficient between TST (+booster) and IGRA was 0.62. **Conclusions:** LTBI is frequent in patients with rheumatic-IMID. IGRA and TST (+booster) show a moderate, fair grade of agreement. Therefore, performing both tests before biological therapy should be highly recommended.

## 1. Introduction

Tuberculosis (TB) remains a major cause of morbidity and mortality worldwide, being the second leading infectious cause of death after COVID-19, ahead of human immunodeficiency virus/acquired immunodeficiency syndrome (HIV/AIDS) [1,2]. In 2020, according to the World Health Organization (WHO), TB was responsible for about 10 million new cases worldwide and around 1.5 million deaths [1].

TB is caused by *Mycobacterium tuberculosis* (MTB). According to the WHO, about 2 to 3 billion people worldwide are currently infected with MTB. However, most of them remain asymptomatic, without developing an active TB disease. This condition is known as latent tuberculosis infection (LTBI) [1]. In fact, only 5% to 15% of LTBI patients will develop active TB during their lifetime. Risk factors associated with progression to TB disease after exposure are low socioeconomic status, work in high-risk settings, malnutrition, diabetes, indoor air pollution, alcohol, HIV/AIDS, autoimmune diseases, and the use of immunosuppressive drugs [3,4]. Immigration may also notably increase the prevalence of LTBI. Among non-US-born persons, the highest LTBI prevalence was estimated in people from Asia and other racial/ethnic groups, who had a 19-fold increased risk compared with US-born persons [3].

Therefore, the accurate diagnosis and prophylactic treatment of LTBI are crucial, particularly in high-risk groups, such as patients with rheumatic immune-mediated inflammatory diseases (rheumatic-IMID) [5]. The prevalence of active TB is double in patients with rheumatic-IMID compared to that in the general population (882/100,000 inhabitants vs 459/100,000 inhabitants) [6]. This risk differs among each rheumatic-IMID when individualized. In rheumatoid arthritis (RA), a significant high risk is observed [7], with a fourfold increased risk noted in a prospective study conducted in Sweden, even in patients not exposed to biological treatments [7,8]. This risk may be even higher in patients with systemic lupus erythematosus (SLE) or Behçet’s disease (BD) [9]. Immunosuppressive drugs, particularly tumor necrosis factor inhibitors (TNFi) and JAK inhibitors (JAKi), are involved in developing active TB [10]. This risk seems to be lower with other non-TNFi biologic drugs. Tumor necrosis factor (TNF) is an essential mediator in the immune response against Mycobacterium tuberculosis infection. Acting in conjunction with interferon-gamma (IFN-γ), it contributes to macrophage activation and its differentiation into specialized cells that form granulomas. The absence of TNF limits the ability to form granulomas, which leads to the progression and dissemination of the infection.

Therefore, MTB reactivation in rheumatic-IMID patients may be due to both the underlying rheumatic-IMID itself and the therapies administered.

Remarkably, no definitive gold standard exists for diagnosing LTBI. The Tuberculin skin test (TST) and interferon (IFN)-γ release assays (IGRAs) are the most frequently used tests [11].

TST is based on a delayed-type hypersensitivity skin reaction to the local injection of purified protein derivative (PPD) or tuberculin. This is the most widely used test for the detection of LTBI, due to its accessibility and low cost. However, this test can show false-positive results in BCG-vaccinated patients and false-negative results in patients with an impaired immune response. The performance of a second TST (booster), one to three weeks later, is recommended to reduce the rate of false-negative results, especially in patients receiving immunosuppressive therapy [12].

IGRA has several advantages compared to TST, as it is not affected by Bacillus Calmette–Guérin (BCG) vaccination, does not require the use of positive and negative controls, requires only one visit, and has a higher sensitivity (84% vs. 67%) and specificity (75% vs. 63%) [13]. Regrettably, the co-occurrence of both rheumatic-IMID and the use of immunosuppressive therapies can negatively affect the accuracy of IGRAs. In fact, immunosuppressive therapy can reduce the IFN-gamma response, which can result in false-negative or indeterminate IGRA results [14,15,16]. 

Interestingly, the percentage of agreement between the TST and IGRA tests was reviewed in nine studies, and four of these studies provided sufficient data to calculate the agreement between the TST and T-SPOT.TB. In fact, Ruan et al. revised all this information in a meta-analysis, concluding that, in rheumatic patients with previous BCG vaccination or under steroid therapy, IGRA would be the best option to identify LTBI [17].

Moreover, neither IGRAs nor TSTs can discriminate between active TB and LTBI and they poorly correlate with the risk of developing the active disease [18]. Furthermore, the sensitivity and specificity of both the IGRA and TST in different rheumatic-IMIDs under immunosuppressive treatments have not been well established.

Taking all this into account, the aims of this study were as follows: (a) to assess the prevalence of LTBI in patients with rheumatic-IMID; (b) to determine the importance of using a TST booster test and/or IGRA test for negative TST results in the detection of LTBI in this population; (c) to perform a literature review of the prevalence of LTBI in different geographical areas and evaluate its relationship with rheumatic-IMID.

## 2. Materials and Methods

### 2.1. Patients and Study Design

We conducted a cross-sectional study in a single university hospital. We included unselected, consecutive patients diagnosed with rheumatic-IMID who underwent a TST and/or IGRA test during a period of five years (2016–2020). These patients were selected from those being prepared for biological therapy in an outpatient consultation. In addition, they were followed up for at least another two years. Furthermore, a complete clinical examination and chest X-ray were performed for all participants to rule out active TB. If the patient had any other type of symptoms, including extrapulmonary symptoms, we continued with further analysis to determine the underlying cause.

### 2.2. Outcome Variables and Clinical Definitions

We obtained the following data from the patients’ clinical records: sex, age, rheumatic-IMID diagnosis, BCG vaccination status, history of previous active TB or LTBI, previous treatments received for active TB or LTBI, chest X-ray findings, and results of the TST, booster, and IGRA.

A TST was performed, read, and interpreted by a trained nurse (VP) in the rheumatology outpatient clinic as a routine screening measure before the start of biological therapy. A TST was performed on all patients, using 0.1 mL of PPD with a concentration of 2 U.T./0.1 mL, with a reading done 72 h afterwards. The TST was considered positive with an induration of more than 5 mm in diameter. If the first TST was negative, a second TST (booster) was performed between 1 and 2 weeks after the first, by injecting the same amount and concentration of PPD as the first time.

The IGRA test used was the QuantiFERON TB Gold Plus (QFT-Plus). The IGRA test was performed using a blood sample. The concentration of IFN-γ (IU/mL) was measured using an automated enzyme-linked immunosorbent assay (ELISA). The results of this test were interpreted using software supplied by the manufacturer, which included a cut-off point for the detection of IFN-γ.

Chest X-rays were obtained for all patients, regardless of whether they had a positive TST and/or IGRA. Active TB was defined according to the European Respiratory Society (ERS)-endorsed official American Thoracic Society/Centers for Disease Control and Prevention/Infectious Diseases Society of America (ATS/CDC/IDSA) clinical practice guidelines on the treatment of drug-susceptible TB, as the presence of clinical findings that suggest active TB, in combination with a chest X-ray showing radiological features consistent with TB [19]. The diagnosis was confirmed using an internationally recommended rapid molecular test and mycobacterial culture. Active TB was treated according to the criteria set by the Spanish Society of Pneumology and Thoracic Surgery (SEPAR) [20].

LTBI was defined by a positive TST and/or IGRA, with no evidence of active TB. LTBI was treated with isoniazid for 9 months, or rifampicin for 4 months when isoniazid was associated with intolerance or toxicity [21].

### 2.3. Data Collection

Data were extracted from the clinical records according to a specifically designed protocol, reviewed for confirmation, and stored in a computerized file. To minimize entry mistakes, all data were double-checked. Finally, the information was stored in a computerized database.

### 2.4. Statistical Analysis

Descriptive statistics (frequency distribution and measures of central tendency and dispersion) were used to analyze the characteristics of the study population. All statistical analyses were performed using the IBM SPSS Statistics software package, v.25. 

The prevalence of LTBI was estimated with 95% confidence intervals (CIs) using the QFT and TST. The concordance between these two tests was evaluated using proportional agreement and Cohen’s kappa coefficient, adjusting for bias and prevalence. The prevalence-adjusted and bias-adjusted kappa (PABAK) provides a measure of the observed agreement, an index of the bias between tests, and an index of the differences between the overall proportions of positive and negative assessments. 

### 2.5. Literature Review

We also performed a literature review in search of previous prevalence studies of LTBI in patients with rheumatic-IMID, which was carried out in August 2022. The aim of this review was to compare the prevalence of LTBI in patients with rheumatic-IMID in different geographical areas.

For this literature review, we searched PubMed, Embase, and the Cochrane Library, with no start date or language restrictions, from January 1st, 2010, to July 31st, 2022, using the keywords (“latent tuberculosis” OR “latent tuberculosis infection” OR “LTBI”) AND (“interferon gamma release assays” OR “Interferon-gamma Release Test” OR “IGRA” OR “QuantiFERON^®^-TB in tube” OR “QFT” OR “T-SPOT.TB”) AND (“tuberculin skin test” OR “tuberculin test” OR “Mantoux test” OR “TST”) AND (“epidemiology”) AND (“rheumatic-IMID” OR “rheumatic inflammatory disease” OR “rheumatoid arthritis” OR “Systemic lupus erythematosus” OR “Behçet’s disease”). Only studies with a sample size of at least 500 participants were included to avoid the selection bias associated with small studies. Meta-analyses, reviews, cost-effectiveness analyses, and studies in non-human models were excluded. We also excluded studies in patients with active or presumptive TB, as well as those that did not report the prevalence of LTBI by IGRA (any version of QFT and/or T-SPOT.TB) or TST tests. In addition, studies targeting high-risk groups (e.g., health care workers, drug users, prisoners, and HIV-positive persons, among others) were discarded to avoid the overestimation of LTBI prevalence during selective screening. We excluded studies that applied interventions that could influence IGRA and TST results, as well as those that selected the population based on specific test results. When the same data appeared in multiple publications, the most complete article, defined as the one with the largest sample size or the most detailed IGRA/TST results, was included. In total, 40 articles met these criteria.

## 3. Results

### 3.1. Clinical Characteristics of the Population Included in this Study

We included 1117 patients, mostly women (741; 62.9%). The mean age was 68 years old.

The main demographic data, rheumatic-IMID distribution, and general TB features of the population included in this study are shown in Table 1.

At the time of the study, a low proportion of patients had been previously vaccinated with BCG (14; 1.3%) or had been diagnosed with active TB (2%). Chest X-rays were normal in most of the patients (108; 96%). Signs of previous TB infection, mostly granulomas, were observed in 13 (1.1%) cases. Cultures were only taken from patients who showed clinical and radiological findings compatible with active tuberculosis (TB), in order to ensure diagnostic accuracy. Only two patients were diagnosed with active TB.

As expected, the most common rheumatic-IMID was rheumatoid arthritis (RA) (*n* = 360; 32.2%), followed by psoriatic arthritis (PsA) (268; 24%), axial spondyloarthritis (SpA) (243; 21.7%), SLE and other connective tissue immune diseases (83; 7.4%), vasculitis/polymyalgia rheumatica (60; 5.4%), sarcoidosis (14; 1.3%), and BD (6; 0.54%). The overall prevalence of LTBI was estimated to be 31.7% (95% CI, 29.74–33.66) in rheumatic IMID, which was determined by calculating the number of cases among the overall population. This prevalence ranged from 14.3% for sarcoidosis to 38.5% for vasculitis. In a heterogeneous group of other rheumatic-IMID, it reached 42.2% of cases.

### 3.2. Data Related to TST and IGRA Examinations

A simple TST examination was performed on all patients. It was positive in 258 patients (23.1%). The booster was performed in the remaining 857 patients with a negative simple TST (two patients were excluded for further analysis because they were finally positive for active TB), and it was positive in 66 patients (7.7%). Therefore, the simple and/or TST booster tests were positive in 324 out of 1117 patients (29%). Results of the TSTs (+booster) and IGRA tests are shown in Table 2.

**Figure 1:**
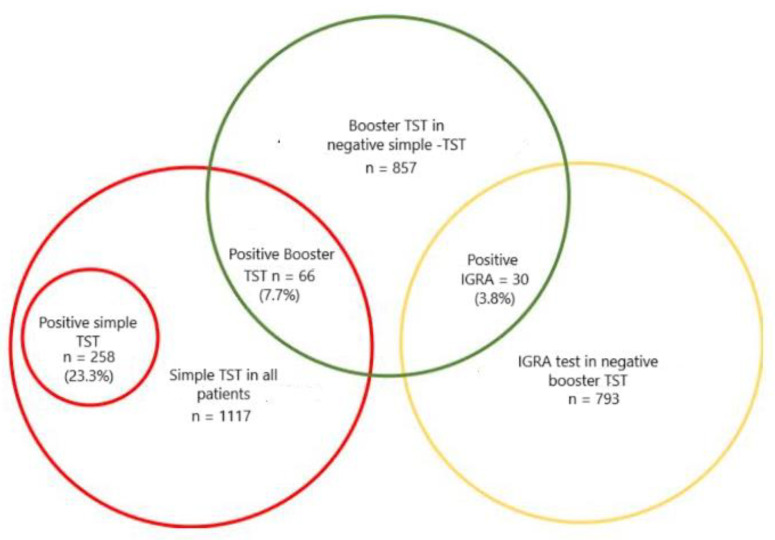
Increased sensitivity in the detection of LTBI in patients with a negative simple TST, an additional booster TST, and an IGRA test.

**Figure 2:**
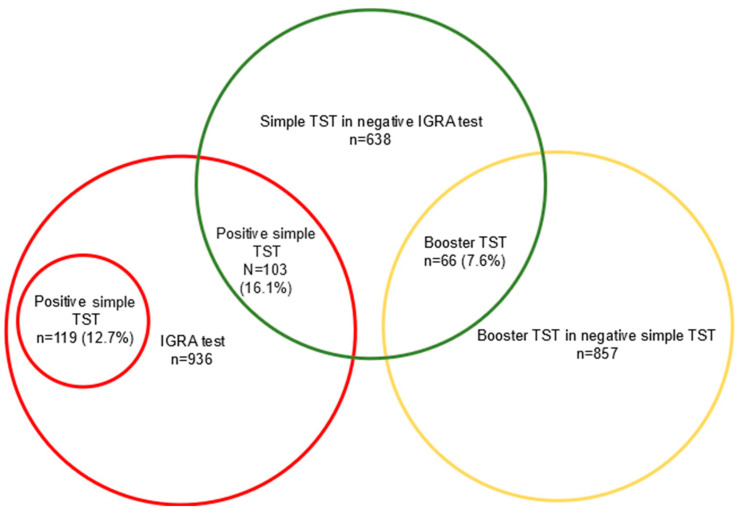
Increased sensitivity in the detection of LTBI in patients with a negative IGRA test, an additional simple TST, and a booster TST.

IGRA was available in 936 of the 1117 patients (83.7%), with 119 cases (12.7%) testing positive, and 817 patients (87.3%) testing negative or indeterminate. Interestingly, the TST (+booster) was positive in 187 (22.9%) out of the 817 patients with a negative or indeterminate IGRA test. Likewise, the IGRA examination was positive in 30 patients (3.8%) out of 793 patients with a negative TST (+booster). The increased performances in patients with negative simple TSTs and IGRA tests, respectively, are shown in Figure 1 and Figure 2.

The IGRA test was not available in 181 cases. In these cases, the TST booster was positive in 130 patients (73.5%). Results of the TST (+ booster) and IGRA tests are shown in Table 2.

### 3.3. Concordance Between the TST and IGRA Examinations

Cohen’s kappa coefficient between the TST (+ booster) and IGRA (QFT-plus) was calculated after adjusting for prevalence and bias. The prevalence- and bias-adjusted kappa (PABAK) was 0.62, which shows a moderate grade of agreement. The concordance between the IGRA and TST examinations are shown in Table 3.

## 4. Discussion

We performed a cross-sectional study at a single university hospital to determine the prevalence of LTBI among patients with rheumatic-IMID in Cantabria, Spain. We also analyzed the performance of IGRAs versus TSTs in patients with rheumatic-IMID prior to starting biological therapy with TNFi.

In this study, we described the prevalence of LTBI in a sample of 1117 patients diagnosed with rheumatic-IMID. Interestingly, 354 patients (31.7%) in our cohort were diagnosed with LTBI using a TST and/or IGRA as screening methods. This result is in line with previous evidence and demonstrates a high prevalence of LTBI in patients with rheumatic-IMID [6]. To consider the results from our study, we have to consider a high prevalence of latent tuberculosis infection in Cantabria. Researchers from the Marqués de Valdecilla Training and Research Institute (IFIMAV) have described the high prevalence (15%−20%) of a genetic variant that increases the susceptibility of developing active pulmonary tuberculosis in people infected with “Mycobacterium tuberculosis” in the population of Cantabria (Spain), according to the National Health Council. Interestingly, the population density in Cantabria is higher than the average for Spain. In fact, the population density of Cantabria doubled between 1900 and 2007 (from 52.5 to 107.7).

To interpret the results in our study, we must consider that the population of Cantabria has a higher population density than the average for Spain. In fact, the population density of Cantabria doubled between 1900 and 2007 (from 52.5 to 107.7). One of the distinctive features of the society is the aging of its population. In fact, the percentage of the population under 15 years old has decreased to a third of people in Cantabria, standing at 12.3% in 2007. This decrease is greater than that experienced at the national level, where the proportion of those under 15 years old was 14.3%, a phenomenon shared by both men and women. While in 1900, the oldest individuals represented only 5.3% of the population of Cantabria (similar to the rest of Spain), in 2007 this group accounted for 18.6% of the entire population. Another aspect that allows us to analyze the transformation of Spain from an eminently agrarian country to a modern one is the improvement in the qualifications of its population. In 1900, 45.2% of the population in Cantabria were illiterate or without formal education, which was more than twenty percentage points lower than that observed at the national level (66.4%). In men, those illiterate or without formal education represented 35.9%, while 53.4% of the female population had no formal qualification. The notable presence of the resident immigrant population in Spain is a recent phenomenon. In the late 1990s, the foreigners in Spain stood at around 3%, while in 2007 they accounted for 6.2%, which is just over half the percentage seen in the rest of Spain (an average of 11.6% for that year). They came mainly from South America, especially from Colombia, Ecuador, and Peru. This information has been provided by the statistics National Institute

Studies regarding the prevalence of LTBI in the general population are shown in Table 4.

In fact, the LTBI prevalence worldwide ranges from 10 cases/100,000 inhabitants to 27.7 cases/100,000 inhabitants. In general, this prevalence is higher in Asian (27.7 cases/100,000 inhabitants) and African (26.6 cases/100,000 inhabitants) countries and lower in the European Union (11.8 cases/100,000 inhabitants), the United States (14 cases/(100,000 inhabitants), and Australia (10.8 cases/100,000 inhabitants) [23,24].

In order to study the prevalence of LTBI in patients with rheumatic-IMID, we performed a literature review. We selected studies performed in several geographical regions worldwide. The results of this literature review are shown in Table 5.

In general, this prevalence ranges from 7% to 67% and varies according to the screening method used, the geographic location, and the population analyzed. Unlike the LTBI prevalence in the general population, the studies conducted in patients with rheumatic-IMID show a higher LTBI prevalence in European countries like France (35.2% prevalence according to TST) or Greece (66.7% prevalence according to TST). In contrast, countries like Brazil (13%), Morocco (7.7%), and Denmark (19%) show lower rates of LTBI. Therefore, our prevalence results align with those reported in countries in our region [35].

The only previous report on LTBI prevalence in rheumatic-IMID conducted in Spain was published in 2012 and shows a prevalence of 13% according to the TST examination. This prevalence is lower than that described in our report. This is likely due to the fact that our study used two LTBI screening tests and due to regional differences inside our country [27].

Regarding specific rheumatic-IMID, 106 patients (29.6%) in our study with RA were diagnosed with LTBI. This prevalence is slightly higher than that described in previous reports, which ranges from 4% to 22%, depending on the method used and the geographic area studied. The prevalence in patients with PsA (24%) or SpA (21.7%) are similar to that described in previous reports, i.e., 2% to 23% for PsA and 5% to 47% for SpA [36,37].

Regarding SLE, the prevalence of LTBI in a study conducted in Spain was around 10%, and for BD, it was 29.3% [27].

Therefore, due to the high prevalence of LTBI in patients with rheumatic-IMID described in this and previous reports, screening for LTBI is strongly recommended in patients with rheumatic-IMID, particularly before starting biological therapy, and especially when TNFi and JAK inhibitors are used.

The screening always involves the exclusion of active TB, and the recommendation of the test of choice (TST or IGRA) may vary according to BCG vaccination [38].

Nonetheless, these tests have also shown false-positive and false-negative results, which are more frequent in patients with rheumatic-IMID receiving immunosuppressive therapy. The IGRA test is altered in immunosuppressed patients receiving GC, biological agents, or an immunosuppressant, and it is still necessary to determine how different diseases can affect the various tests. This can lead to varying prevalence rates of LTBI depending on the underlying disease. For now, the Centers for Disease Control and Prevention (CDC) recommends using both tests in combination for the detection of LTBI, due to the different causes of false-positive reactions for the TST; these are mainly related to previous TB vaccination with the Bacillus Calmette–Guérin (BCG) vaccine and infection with nontuberculous mycobacteria (mycobacteria other than *M. Tuberculosis*) [39]. Nevertheless, these findings highlight the need for a diagnostic test with improved sensitivity [21].

Finally, we showed that some patients who had a negative or indeterminate result on the IGRA test or TST could have a positive result in the other examination. This highlights the importance of performing both tests for all these patients. Otherwise, a patient with a false-negative result in one test might start receiving biologics without prior chemoprophylaxis, while they are at risk of developing active TB. Therefore, we reaffirmed the importance of performing both examinations before starting biological therapy for patients with rheumatic-IMID.

However, our study has some limitations. In fact, not all the patients included in our study were tested for IGRA. Additionally, the issue of false positives and false negatives in both tests, especially TST, could indicate a relative increase in cases and lead to the incorrect treatment of patients, i.e., those with a false positive due to a previous vaccination, as well as those with false negatives in both tests, related to immunosuppressive treatment, which was information not collected in our study. We also want to emphasize that these patients were selected from those being prepared for biological therapy, so they may not be representative of the disease as a whole, as the disease activity, severity, and previous treatment may affect the prevalence of latent TB tests. Since we did not collect information related to previous immunosuppressive treatment, we cannot explicitly state whether patients were on treatment or not. Since the treatment status is unknown, related statements about the data presented should be interpreted accurately. Another limitation of our study is the influence of cases from high TB-burden countries on the prevalence in our population.

Finally, one of the most important limitations of this study is that the statistical analysis is relatively basic. This may affect the ability to detect complex associations and increase the risk of bias in the results. It also limits the potential to generalize the findings to other populations, among other inherent restrictions of this type of analysis that could influence our results.

## 5. Conclusions

In summary, this cross-sectional study has shown a high prevalence of LTBI in patients with rheumatic-IMID in our setting. This emphasizes the need for a provocative preliminary assessment of LTBI before administering biological therapy. We also conclude that preliminary TB screening must be conducted using both TST and IGRA as complementary methods in individuals with suspected LTBI, as both tests can yield false-negative results, especially in patients with rheumatic-IMID receiving immunosuppressive therapy. This study also highlights the need to develop new diagnostic tools to detect LTBI.

## Figures and Tables

**Table 1:** Baseline general features of patients included in this study.

Rheumatic-IMID	Cases *n* (%)	Age, Mean ± SD, Years	Gender Female, *n* (%)	Previous BCG Vaccination *n* (%)	Previous Active TB *n* (%)	Residual TB *n* (%)	LTBI Prevalence *n* (%)
RA	360 (32.2)	59.4 ± 13.9	278 (77.2)	2 (0.6)	7 (1.9)	10 (2.8)	106 (29.4)
PsA	268 (24)	50.8 ± 11.9	155 (57.8)	5 (1.9)	2 (0.7)	0	87 (32.5)
SpA	243 (21.7)	45.7 ± 12.8	126 (51.9)	4 (1.6)	5 (2.1)	1 (0.4)	79 (32.5)
SLE	36 (3.2)	47.2 ± 12.9	33 (91.7)	1 (2.7)	1 (2.7)	0	9 (25)
Other connective tissue diseases	47 (4.2)	55.7 ± 13.9	36 (76.6)	1 (2.1)	1 (2.1)	0	13 (27.7)
Vasculitis	39 (3.5)	65.3 ± 15.5	26 (66.7)	0	4 (10.3)	0	15 (38.5)
PMR	21 (1.9)	69.6 ± 10.1	16 (76.2)	0	0	0	6 (28.6)
Sarcoidosis	14 (1.3)	51.6 ± 13.4	7 (50)	0	0	0	2 (14.3)
BD	6 (0.54)	45.8 ± 07.9	4 (66.7)	0	1 (16.7)	0	2 (33.3)
Others	83 (7.4)	50.4 ± 15.9	60 (7.2)	1 (1.2)	2 (2.4)	2 (2.4)	35 (42.2)
TOTAL	1117	53.0 ± 15.0	741 (62.9)	14 (1.3)	23 (2)	13 (1.1)	354 (31.7)

Abbreviations (in alphabetical order): BCG: Bacillus Calmette-Guérin, BD: Behçet’s disease, LTBI: latent tuberculosis infection, PMR: polymyalgia rheumatica, PsA: psoriatic arthritis, RA: rheumatoid arthritis, rheumatic-IMID: rheumatic immune-mediated inflammatory diseases, SD: standard deviation, SLE: systemic lupus erythematosus, SpA: axial spondyloarthritis, TB: tuberculosis.

**Table 2:** Results of the TST (+ booster) and IGRA examinations.

		IGRA (QFT-Plus)				
		Positive *n* (%)	Negative *n* (%)	Indeterminate *n* (%)	Unavailable *n* (%)	Total N
**TST** **(+booster)**	Positive	89 (74.8)	142 (22.1)	45 (25.7)	48 (26.5)	324
	Negative	30 (25.2)	500 (77.9)	130 (74.3)	133 (73.5)	793
	Total	119	642	175	181	1117

Abbreviations (in alphabetical order): IGRA (QFT-Plus): interferon-gamma release assay (QuantiFERON-TB Gold Plus), TST: tuberculin skin tests.

**Table 3:** Concordance between Quantiferon and the TST (+ booster).

	TST (+Booster)		
Quantiferon	**Negative**	**Positive**	
Negative	537	103	640 (84%)
Positive	41	77	118 (16%)
Negative + positive	578 (76%)	180 (24%)	758 (100%)
Kappa index	0.409 (0.326−0.483)		
**Observed Agreement**			
Specific agreement + (%)		88 (86−90)	
Specific agreement–(%)		52 (46−57)	
Global agreement (%)		81 (78−84)	
**Observed Disagreement**			
Quantiferon + TST (+ booster)–(%)		5 (4−7)	
Quantiferon–TST (+ booster) + (%)		14 (12−17)	
Global disagreement (%)		19 (16−22)	
Bias index (%)		8 (5−11)	
Bias-adjusted Kappa		0.419 (0.336−0.503)	
Prevalence index		61%	
**Prevalence- and Bias-adjusted Kappa**		**0.620 (0.559−0.681)**	

Abbreviations (in alphabetical order): IGRA (QFT-Plus): interferon-gamma release assay (QuantiFERON-TB Gold Plus), TST: tuberculin skin tests.

**Table 4:** Distribution of LTBI in the general population according to geographic regions worldwide.

Author, Year (Ref. in Text)	Region	Diagnostic Criteria- Study Period	Total Number of Cases	Prevalence Rate in General Population (Over 100,000)	Active TB Incidence, Rates (per 100,000 Population) in 2020 According to WHO
Mancuso JD, et al. (2021) [22]	United States	QFT 2011−2012	14,000,000	14 (11.9–16.5)	2.4
Cohen A, et al. (2019) [23]	Region of the Americas (AMR)	TST and IGRAs 2005 and 2018	190,052,252	13.5 (9.7–17.2) 13.7 (11.0–16.3)	9.9
Cohen A, et al. (2019) [23]	Eastern Mediterranean	TST and IGRAs 2005 and 2018	130,680,214	21.1 (17.4–24.8) 24.0 (19.4–28.5)	2.3
Cohen A, et al. (2019) [23]	European Region (EUR)	TST and IGRAs 2005 and 2018	125,126,219	11.8 (8.6–15.0) 12.2 (9.8–14.5)	12
Cohen A, et al. (2019) [23]	South East Asian Region (SEA)	TST and IGRAs 2005 and 2018	524,749,757	27.7 (23.6–31.8) 36.0 (25.3–46.7)	1.9
Cohen A, et al. (2019) [23]	African Region (AFR)	TST and IGRAs 2005 and 2018	250,281,392	26.6 (23.0–30.2) 33.6 (24.4–42.9)	24
Cohen A, et al. (2019) [23]	Western Pacific Region (WPR)	TST and IGRAs 2005 and 2018	578,305,937	20.3 (15.0–25.7) 20.7 (16.8–24.5)	2.1
Ding C, et al. (2022) [24]	Japan	TST 1990–2019	22,605,381	17.82/15.72	11.5
Ding C, et al. (2022) [24]	Australia	TST 1990–2019	2,617,835	10.8/9.88	5.5

Abbreviations (in alphabetical order): AFR: African Region, AMR: Region of the Americas, EUR: European Region, IGRA (QFT): interferon-gamma release assay (QuantiFERON-TB Gold), SEA: South East Asian Region, TB: tuberculosis, TST: tuberculin skin tests, WPR: Western Pacific Region, WHO: World Health Organization.

**Table 5:** Prevalence of LTBI according to underlying rheumatic-IMID worldwide.

Author, Year (Ref. in Text)	Region	Diagnostic Criteria Study Period	Prevalence in Rheumatic-IMID (%)	Prevalence in RA (%)	Prevalence in PsA (%)	Prevalence in AS (%)	Prevalence in SLE	Prevalence in BD
Soborg B, et al. (2009) [25]	Denmark	TST and IGRA 2005 to March 2007	19% 7%	20% 9%		28% 5%		
Chang B, et al. (2011) [26]	Korea	TST or IGRA test (2007-July 2009)	35% 30%	22% 31%		47% 29%		
Mínguez S, et al. (2012) [27]	Spain	TST, T-SPOT.TB and QTF-plus 2008−2010	13% 20% 17%	16% 22% 22%				
Mariette X, et al. (2012) [28]	France	TST, T-SPOT.TB and QTF-plus (2011)	35.2% 15.1% 9.9%					
Miras M, et al. (2014) [29]	Spain	TST and T-SPOT.TB 2009−2012					7% 5%	
Gomes CM, et al. (2015) [30]	Brazil	TST	20.6%	12.8%	18.8%	37.6%		
Perifanou D, et al. (2018) [31]	Greece	TST and IGRA test 2008−2010	66.7% 31%	15%		21%		
Anton C, et al. (2019) [6]	Brazil	TST	13%	4%	23%	26%		
Meriem S, et al. (2019) [32]	North of Tunisia	TST and IGRA test 2015−2017	45.7% 21.9%					
Oulkadi L, et al. (2021) [33]	Morocco	TST and IGRA test 2017−2021	7.7% 15.4%	15.9%%	2.4%	24.8%		
Shen Y, et al. (2021) [34]	China	T-SPOT.TB 2012−2017						29.3%
Present series	Spain	TST and QuantiFERON TB Gold Plus	31.7%	29.4%	32.5%	32.5%	25%	33.3%

Abbreviations (in alphabetical order): AS: ankylosing spondylitis, BD: Behçet’s disease, IGRA: interferon-gamma release assay, RA: rheumatoid arthritis, PsA: psoriatic arthritis, QFT-plus: QuantiFERON TB Gold Plus, rheumatic-IMID: rheumatic immune-mediated inflammatory diseases, SLE: systemic lupus erythematosus, TST: tuberculin skin test.

## Data Availability

Data is available upon reasonable request by any qualified researchers who engage in rigorous, independent scientific research, and will be provided following the review and approval of a research proposal and Statistical Analysis Plan (SAP) and the execution of a Data Sharing Agreement (DSA). All data relevant to the study are included in this article.
